# TAK-442, a Direct Factor Xa Inhibitor, Inhibits Monocyte Chemoattractant Protein 1 Production in Endothelial Cells via Involvement of Protease-Activated Receptor 1

**DOI:** 10.3389/fphar.2018.01431

**Published:** 2018-12-04

**Authors:** Emiko Shinozawa, Masaharu Nakayama, Yoshimi Imura

**Affiliations:** Research, Takeda Pharmaceutical Company Limited, Fujisawa, Japan

**Keywords:** factor Xa, thrombin, monocyte chemoattractant protein 1, endothelial cells, protease-activated receptor 1

## Abstract

Oral blood coagulation inhibitors and their receptors, such as factor Xa (FXa), thrombin, and the thrombin receptor protease-activated receptor 1 (PAR1), are entered into clinical trials for acute coronary syndrome therapy; however, the results obtained so far are different for each drug. The underlying mechanisms of the results have not been fully investigated. We studied the *in vitro* anti-inflammatory effects of the selective FXa inhibitor TAK-442 on human endothelial cells, with comparing those of the selective thrombin inhibitor melagatran and the PAR1 antagonist vorapaxar. In human umbilical vein endothelial cells, FXa-increased production of monocyte chemoattractant protein 1 (MCP-1), a key inflammatory mediator, was inhibited by TAK-442 but not melagatran, and was also remarkably suppressed by vorapaxar. As thrombin did, FXa increased calcium mobilization in PAR1-overexpressed Chinese hamster ovary cells, which was selectively inhibited by TAK-442 and vorapaxar. We therefore confirmed the inhibitory effect of TAK-442 in endothelial MCP-1 production and the PAR1 intervention in the response. Our results suggest that TAK-442 may have anti-inflammatory potential in addition to its anti-thrombotic effects.

## Introduction

Blood coagulation factor Xa (FXa) and thrombin, along with other coagulation factors, play a crucial role in the coagulation cascade and also reportedly induce pro-inflammatory responses in various types of cells (Strukova, 2006). In particular, FXa and thrombin enhance production of chemoattractant proteins, such as monocyte chemoattractant protein 1 (MCP-1) and interleukin-8, in endothelial cells. These responses have been reported to be mediated by their receptor protease-activated receptors 1 (PAR1), an antagonist of which is also known as an anti-platelet drug. These findings are ones which support the activated vascular coagulation-inflammatory circuit, which can cause the development of atherosclerosis, and consequently acute coronary syndromes (ACS) (Gerszten et al., 1999; Viles-Gonzalez et al., 2004). Therefore, an inhibition of the coagulation and inflammatory pathways is considered to be efficacious for the treatment of ACS. Especially, inhibitors of FXa and thrombin are expected to be effective via anti-inflammatory effects in addition to their anti-thrombotic effects; however, some inhibitors showed efficacy in patients with ACS while others did not, and the underlying mechanisms are not fully investigated. TAK-442 is an oral direct FXa inhibitor. The safety and tolerability of TAK-442 were also tested in the patients following ACS. In consequence, there was no significant difference in the primary endpoint of the study in addition to its efficacy (Goldstein et al., 2014). However, little is elucidated about the potential of TAK-442 to affect inflammation and to exert a specific effectiveness. We studied and here report the anti-inflammatory effect of TAK-442 on production of a key inflammatory mediator, MCP-1, in human umbilical vein endothelial cells (HUVECs).

## Materials and Methods

### Materials

The selective FXa inhibitor TAK-442, 1-(1-{(2S)-3-[(6-chloro–2-naphthyl) sulfonyl]-2-hydroxypropanoyl}piperidin-4-yl)tetra hydropyrimidin-2(1H)-one, the selective thrombin inhibitor melagatran, N-((1R)-2-{(2S)–2-[({4-[amino(imino)methyl]benzyl}amino)carbonyl]azetidin-1-yl}-1–cyclohexyl-2-oxoethyl) glycine, and the selective PAR1 antagonist vorapaxar, ethyl(9-{(E)–2-[5-(3-fluorophenyl)pyridin-2-yl]vinyl}-1-methyl-3-oxododecahydronaphtho[2,3-c]furan-6-yl)carbamate, were synthesized at Takeda Pharmaceutical Co., Ltd. (Osaka, Japan). Human blood-derived FXa and thrombin were purchased from Calbiochem (EMD Millipore, Merck KGaA, Darmstadt, Germany) and Sigma-Aldrich Japan (Tokyo, Japan), respectively.

### Measurement of MCP-1

Human umbilical vein endothelial cells (pooled) were purchased from Lonza Japan (Tokyo, Japan) and maintained in endothelial cell growth medium-2 (EGM-2; Lonza Japan) containing 2% fetal bovine serum at 37°C in an atmosphere of 5% CO_2_. Subconfluent HUVECs at passage 4 were treated with the media containing each coagulation factor, FXa and thrombin, at final concentrations up to 1 U/mL. Following a 20-h incubation period, the supernatant was pooled and stored at -80°C until measurement. Inhibition studies were carried out with the addition of each inhibitory or antagonistic compound 1 h prior to FXa or thrombin addition. The compounds were dissolved with dimethyl sulfoxide (DMSO). The final concentration of DMSO in culture medium was adjusted to 0.1%. Levels of MCP-1 were determined with a sandwich enzyme-linked immunosorbent assay (ELISA) method using the Human CCL2/MCP-1 Quantikine ELISA kit (R&D Systems, Inc., Minneapolis, MN, United States). Briefly, the supernatant sample or MCP-1 standard solution was added to a 96-well plate pre-coated with goat anti-human MCP-1 monoclonal antibodies and incubated for 2 h at room temperature. Following incubation, wells were washed and treated with horseradish peroxidase-conjugated antibody specific for MCP-1 for 1 h at room temperature. Chromogenic substrate was added to each well and the results were quantified after the addition of reaction stop solution by measuring the optical density at 450 nm using an automated plate reader. A standard curve was constructed to calculate MCP-1 levels in samples. The concentration of each compound required to suppress the increased MCP-1 production by 50% (IC_50_) was determined.

### Measurement of Intracellular Calcium Using the FLIPR

Chinese hamster ovary (CHO-K1) cells stably expressing human PAR1 (hPAR1/CHO-K1 cells) were seeded at a density of 3,000 cells/well into 96-well plates with clear bottoms (Corning Inc., New York, NY, United States) and cultured for 20–24 h at 37°C in an atmosphere of 5% CO_2_. Following incubation, cells were washed and incubated with the assay buffer containing 2.5 μg/mL Fluo-3AM (Dojindo Laboratories, Kumamoto, Japan), 0.08% Pluronic F-127, and 2.5 mM probenecid for 1 h at 37°C. The cells were washed twice and resuspended in the assay buffer. The plate was placed into FLIPR (Molecular Devices Japan K.K., Tokyo, Japan) to monitor cell fluorescence before and after the addition of FXa, thrombin, or PAR1 agonist peptide SFLLRN-NH2. For inhibition studies, FXa and thrombin were used at concentrations of 0.03 and 0.003 U/mL, respectively, and SFLLRN-NH2 at 3 nM, such that the change in fluorescence intensity caused by each agonist was approximately 15,000 fluorescence counts. Each of the compounds, TAK-442, melagatran, or vorapaxar, was pre-incubated with cells for 10 min prior to experiments. The concentration of compound required to suppress the intracellular calcium concentration by 50% (IC_50_) was determined.

### Statistical Analysis

Data are presented as mean ± standard error of the mean (SEM). The statistical significance was determined by the Student’s *t*-test or one-tailed Williams’ test to evaluate the significance for each treated group compared to the control group, following one-way analysis of variance (ANOVA) model (for multiple groups). A *P*-value of ≤ 0.05 and ≤ 0.025 was considered significant for the Student’s *t*-test and one-tailed Williams’ test, respectively. The IC_50_ value was calculated using a non-linear logistic model.

## Results

### MCP-1 Production by FXa and Thromobin in HUVECs

**FIGURE 1:**
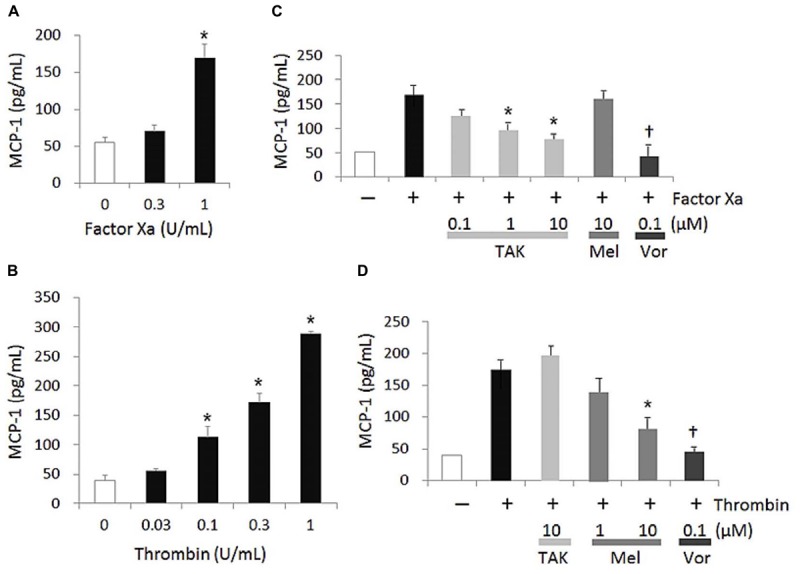
FXa- and thrombin-induced MCP-1 production in HUVECs. MCP-1 concentration was determined in cell supernatant collected 20 h after the addition of the agonist, FXa **(A)** or thrombin **(B)**. In inhibition studies **(C,D)**, each inhibitor was added 1 h prior to the addition of the agonist and effects of TAK-442 (TAK), melagatran (Mel), and vorapaxar (Vor) on MCP-1 productions induced by 1 U/mL FXa **(C)** and 0.3 U/mL thrombin **(D)** were measured. Data are shown as mean ± SEM (*n* = 3). ^∗^*P* ≤ 0.025 compared with the control value of cells treated without FXa or thrombin (one-tailed Williams’ test) **(A,B)**. ^∗^*P* ≤ 0.025 and ^†^*P* ≤ 0.05 compared with the control value of cells treated with FXa or thrombin and no inhibitor (one-tailed Williams’ test and Student’s *t*-test, respectively, following ANOVA) **(C,D)**.

Both FXa and thrombin increased MCP-1 secretion from HUVECs in a concentration dependent manner (Figures 1A,B). The significant elevation of MCP-1 production was observed upon stimulation with FXa and thrombin at 1 and more than 0.1 U/mL, respectively. For inhibition studies, FXa and thrombin were used at 1 and 0.3 U/mL, respectively, which were concentrations required to achieve similar MCP-1 expression levels (Figures 1C,D). Under this condition, FXa- and thrombin-induced MCP-1 productions were significantly reduced by TAK-442 at more than 1 μM and melagatran at 10 μM, respectively, and completely inhibited by vorapaxar at 0.1 μM. TAK-442 and melagatran, both at 10 μM, did not affect thrombin- and FXa-induced MCP-1 productions, respectively. The IC_50_ value of TAK-442 in the FXa-induced response was 0.34 μM.

### Calcium Mobilization in PAR1-Overexpressed hPAR1/CHO-K1 Cells

**FIGURE 2:**
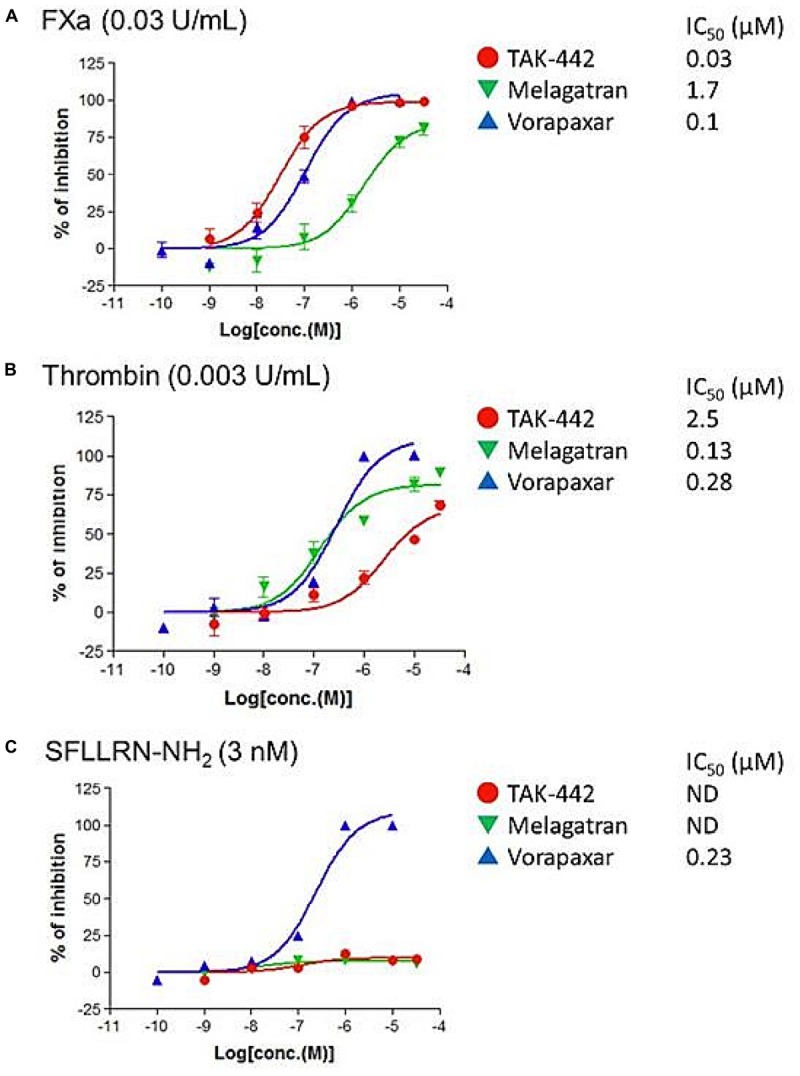
Effects of TAK-442, melagatran, and vorapaxar on the intracellular calcium ion concentration ([Ca^2+^]i) induced by FXa, thrombin, and SFLLRN-NH2 in human PAR1-transfected Chinese hamster ovary (hPAR1/CHO-K1) cells. Calcium signal was recorded after the addition of FXa (0.03 U/mL) **(A)**, thrombin (0.003 U/mL) **(B)**, or PAR1 agonist peptide SFLLRN-NH2 (3 nM) **(C)** using FRIPR. Each inhibitor, TAK-442, melagatran, or vorapaxar, was pre-incubated with the cells for 10 min before the treatment with each agonist. Data are expressed as the percentage inhibition of calcium signal obtained after the addition of agonist in inhibitor-treated wells (*n* = 4) compared with control wells (no inhibitor added). The drug concentration need to suppress the [Ca^2+^]i by 50% (IC_50_) was determined.

In the PAR1-transfected hPAR1/CHO-K1 cells, both FXa- and thrombin-increases in [Ca^2+^]i were suppressed by TAK-442 and melagatran, respectively, and were almost equally suppressed by vorapaxar (Figures 2A,B). At higher concentrations, TAK-442 and melagatran affected thrombin- and FXa-increased [Ca^2+^]i, respectively. The elevation in [Ca^2+^]i in the presence of SFLLRN-NH2 was inhibited by vorapaxar but not TAK-442 or melagatran (Figure 2C).

## Discussion

In the present study, as we expected, TAK-442 significantly inhibited the MCP-1 production induced by FXa, as melagatran affected that induced by thrombin. First, we confirmed that FXa and thrombin enhanced MCP-1 secretion from HUVECs in a concentration-dependent manner, which is almost in line with previous studies *in vitro* (Marin et al., 2001; Busch et al., 2005; Mhatre et al., 2016). Although exact physiological concentrations of FXa and thrombin in plasma are unknown, the concentrations of FXa and thrombin used in our HUVEC assay can be thought to be at least locally attainable under physiological conditions (Putnam, 1984; Perry and Pasi, 1999). Under these assay conditions, we studied the effect of TAK-442, which displays good selectivity for FXa over other human serine proteases (greater than 500-fold) (Fujimoto et al., 2010). The concentration range of TAK-442 used in the assay was expected to be attained *in vivo*; specifically, in the previous studies, a doubling of the clotting time parameter prothrombin time was observed in a venous thrombosis model in rats orally administrated with TAK-442, and also TAK-442 showed *in vitro* the prothrombin time doubled in rat plasma at approximately 0.5 μM (Kawamura et al., 2010; Konishi et al., 2010). TAK-442 was also shown to have similar *in vitro* prothrombin time-prolonging activities in plasma of both rat and human (Kawamura et al., 2010), suggesting that the IC_50_ value of TAK-442 obtained in our HUVEC assay could be also be preferable to exert its anticoagulant effect in a clinical situation. In other studies already reported, FXa-, thrombin-, and plasma-induced endothelial inflammatory gene expressions were suppressed by direct oral anticoagulants such as another FXa inhibitor rivaroxaban, and of the reported genes, MCP-1 was one of those most strongly related (Ellinghaus et al., 2016; Seki et al., 2017). Therefore, besides the previous findings, our study confirmed the anti-inflammatory effect of TAK-442, and reconfirmed that endothelial MCP-1 production was induced by FXa independently of thrombin, through a comparative study using melagatran, which is a selective inhibitor for thrombin over other serine proteases (greater than 1000-fold) except for trypsin (Park et al., 2013).

We also showed that the both FXa- and thrombin-increased endothelial MCP-1 secretions from HUVECs were almost inhibited by the PAR1 antagonist, vorapaxar. Vorapaxar was reported to be a selective PAR1 antagonist, which was inactive in the PAR2, PAR3 binding, and PAR4 functional assays (Chackalamannil et al., 2008). FXa-induced PAR1 activation, including endothelial one, was shown by others to be independent of thrombin (Riewald et al., 2001; Camerer et al., 2002). PAR2 was also reported to have an interaction with FXa in endothelial pro-inflammatory responses, and protective and permeability barrier function (Feistritzer et al., 2005; Daubie et al., 2006), and also in suppression of pro-inflammatory cytokine production in mononuclear cells and macrophages (Gleeson et al., 2014). Altogether, the PARs, depending on the conditions of their activation prevailing in each cell type, are considered to mediate dual responses: not only anti-inflammatory but also pro-inflammatory responses, one resulting in the regulation of endothelial secretory activity (Ossovskaya and Bunnett, 2004; De Ceunynck et al., 2018). Besides, endothelial cytokine secretion is reportedly mediated by the inter-epidermal growth factor sequence Leu-83 to Leu-88 of FXa via binding to the effector cell protease receptor-1, another endothelial FXa receptor (Altieri, 1994; Papapetropoulos et al., 1998; Senden et al., 1998). The remaining FXa-induced MCP-1 production when treated with TAK-442 in Figure 1C may be the result of both those contradictory roles of PARs and other signal transductions. Nevertheless, overall, from our results in Figure 1, it was thought that as thrombin did, FXa used mainly PAR1 in its signal transduction, resulting in endothelial MCP-1 production, under the conditions of the present study. We, therefore, further sought to confirm the PAR1 intervention in the anti-inflammatory effect of TAK-442, using intracellular calcium mobilizing system with hPAR1/CHO-K1 cells. We first measured the [Ca^2+^]i in untransfected CHO-K1 cells and detected only weak signals upon stimulation with FXa, thrombin, and PAR1 agonist peptide SFLLRN-NH2 (data not shown). This confirmed the absence of any route other than PAR1 involved in our [Ca^2+^]i mobilization system. Then, in the PAR1-transfected hPAR1/CHO-K1 cells, we found that the both FXa- and thrombin-increases in [Ca^2+^]i were preferentially suppressed upon treatment with their respective inhibitors. These results suggest that FXa and thrombin can activate PAR1 signaling independently. In addition, it was suggested that in the signal transduction, both FXa and thrombin neither exhibit any competitive antagonistic action against PAR1 ligands nor exert a direct effect on PAR1 downstream factors.

Taken together, we confirmed the inhibitory effect of TAK-442 on endothelial MCP-1 production and highlighted the role of PAR1 signaling in the response. Although there was no response or pathway identified, which was unique to TAK-442 or FXa and different from one involving thrombin or PAR1, our finding for another inhibitor TAK-442 will be an additional related piece of evidence in interpreting the coagulation-inflammation cycle. Further investigation requires elucidating the anti-inflammatory efficacy of TAK-442 in physiological settings beyond cell culture models; however, our results proposed that TAK-442 may still have potential to be therapeutically effective when the relation between coagulation and inflammation pathways is activated, including with ACS.

## Author Contributions

ES and MN designed and performed the experiments, interpreted data, and wrote the manuscript. YI designed the experiments, interpreted data, and wrote the manuscript.

## Conflict of Interest Statement

All of the authors were employed by Takeda Pharmaceutical Company Limited at the time of this study. The funders had no role in study design, data collection and analysis, decision to publish, or preparation of the manuscript.The reviewer AR and handling Editor declared their shared affiliation.
